# Supplementary material for: ProteinShader: illustrative rendering of macromolecules
Source: BMC Struct Biol. 2009 Mar 30;9:19. doi: 10.1186/1472-6807-9-19 (PMC2672931; doi:10.1186/1472-6807-9-19)
Supplement: Additional file 1 — ProteinShader program without source code. This compressed file contains the complete ProteinShader program including associated libraries, but no source code. A README.txt file gives an overview of the ProteinShader distribution, and the index.html file in the help subdirectory has directions on getting started with the program as well as a set of tutorials. [file 1472-6807-9-19-S1.zip › ProteinShader-beta-0_9_4-binary/help/api/org/proteinshader/structure/exceptions/class-use/InvalidRegionException.html]

Uses of Class org.proteinshader.structure.exceptions.InvalidRegionException (ProteinShader API)


|  |  |  |
| --- | --- | --- |
| **Overview** \| **Package** \| **Class** \| **Use** \| **Tree** \| **Deprecated** \| **Index** \| **Help** | |  |
| PREV   NEXT | **FRAMES**    **NO FRAMES**     **All Classes** |  |


---


## **Uses of Class org.proteinshader.structure.exceptions.InvalidRegionException**

| Packages that use InvalidRegionException | |
| --- | --- |
| **org.proteinshader.structure** | Holds the classes that store information from a Protein Data Bank file: Structure, Model, Chain, AminoAcid, Heterogen, Water, Atom, Bond, Helix, BetaStrand, Loop, *etc*. |

Uses of InvalidRegionException in org.proteinshader.structure

| Methods in org.proteinshader.structure that throw InvalidRegionException | |
| --- | --- |
| `BetaStrand` | `Chain.addNewBetaStrand(String betaStrandID, String betaStrandNumber, String sheetID, String startResidueID, String endResidueID, int sense, int strandsInSheet)`             Creates a new BetaStrand with the BetaStrand ID given as an argument, adds the BetaStrand to the Chain's collection of BetaStrands, and returns a reference to the new BetaStrand. |
| `void` | `Structure.addNewBetaStrand(String chainID, String betaStrandID, String betaStrandNumber, String sheetID, String startResidueID, String endResidueID, int sense, int strandsInSheet)`             The BetaStrand will be added to the equivalent Chain on each Model, but this addition will only work if the sequence of Residues that the BetaStrand refers to have already been added. |
| `void` | `Model.addNewBetaStrand(String chainID, String betaStrandID, String betaStrandNumber, String sheetID, String startResidueID, String endResidueID, int sense, int strandsInSheet)`             The BetaStrand will be added to the Chain only if the sequence of Residues that the BetaStrand refers to has already been added. |
| `Helix` | `Chain.addNewHelix(String helixID, String serialNo, String startResidueID, String endResidueID, HelixEnum type)`             Creates a new Helix with the Helix ID given as an argument, adds the Helix to the Chain's collection of Helices, and returns a reference to the new Helix. |
| `void` | `Structure.addNewHelix(String chainID, String helixID, String serialNo, String startResidueID, String endResidueID, HelixEnum type)`             The Helix will be added to the equivalent Chain on each Model, but this addition will only work if the sequence of Residues that the Helix refers to have already been added. |
| `void` | `Model.addNewHelix(String chainID, String helixID, String serialNo, String startResidueID, String endResidueID, HelixEnum type)`             The Helix will be added to the Chain only if the sequence of Residues that the Helix refers to has already been added. |
| `Loop` | `Chain.addNewLoop(String loopID, String startResidueID, String endResidueID)`             Creates a new Loop with the Loop ID given as an argument, adds the Loop to the Chain's collection of Loops, and returns a reference to the new Loop. |

Constructors in org.proteinshader.structure that throw InvalidRegionException

`BetaStrand(String betaStrandID,
String betaStrandNumber,
String sheetID,
String startResidueID,
String endResidueID,
Chain chain,
int sense,
int strandsInSheet)`
  
          Constructs a BetaStrand.

`Helix(String helixID,
String serialNo,
String startResidueID,
String endResidueID,
Chain chain,
HelixEnum type,
HelixShapeEnum shape)`
  
          Constructs a Helix.

`Loop(String loopID,
String startResidueID,
String endResidueID,
Chain chain)`
  
          Constructs a Loop.

`Region(String startResidueID,
String endResidueID,
Chain chain)`
  
          Constructor for use by subclasses.

---


|  |  |  |
| --- | --- | --- |
| **Overview** \| **Package** \| **Class** \| **Use** \| **Tree** \| **Deprecated** \| **Index** \| **Help** | |  |
| PREV   NEXT | **FRAMES**    **NO FRAMES**     **All Classes** |  |


---

# *Copyright © 2007-2008*
